# The Characterization of *Enterococcus* Genus: Resistance Mechanisms and Inflammatory Bowel Disease

**DOI:** 10.1515/med-2020-0032

**Published:** 2020-04-03

**Authors:** Michaela Růžičková, Monika Vítězová, Ivan Kushkevych

**Affiliations:** 1 Department of Experimental Biology, Faculty of Science, Masaryk University, Kamenice 753/5, 625 00 Brno, Czech Republic

**Keywords:** *Enterococcus faecium*, *Enterococcaceae*, vancomycin, resistance, antibiotics, nosocomial diseases

## Abstract

The constantly growing bacterial resistance against antibiotics is recently causing serious problems in the field of human and veterinary medicine as well as in agriculture. The mechanisms of resistance formation and its preventions are not well explored in most bacterial genera. The aim of this review is to analyse recent literature data on the principles of antibiotic resistance formation in bacteria of the *Enterococcus* genus. Furthermore, the habitat of the *Enterococcus* genus, its pathogenicity and pathogenicity factors, its epidemiology, genetic and molecular aspects of antibiotic resistance, and the relationship between these bacteria and bowel diseases are discussed. So-called VREfm – vancomycin resistant *Enterococcus faecium* and its currently rapidly growing resistance as well as the significance of these bacteria in nosocomial diseases is described.

## 1 Introduction

Enterococci have become the second most common agents of nosocomial diseases due to their constantly growing resistance and nowadays are ranked right after staphylococci. It was discovered and later proved, that present multi-resistant *E. faecium* belongs to a different taxon than the original strains isolated from animals. This separation must have happened around 75 years ago and is being connected to antibiotic usage in clinical practice. This clade can be distinguished by its increased number of mobile genetic elements, metabolic alternations and hypermutability [1]. All of these attributes led to the development of a flexible genome, which is now able to easily adapt to the changes of the surroundings [2,3].

It is also necessary to mention some basic information about morphological diversity, physiological and biochemical characteristics and taxonomy in order to understand the resistance mechanisms completely. Biochemical features must especially be mentioned since they play a huge role in the high increase of resistance in these bacteria.

It is also crucial to describe the natural habitats of enterococci and their pathogenicity since these factors are really related to each other. When in their natural habitat - such as soil or gastrointestinal tract – enterococci are quite harmless, but in the presence of a hospital environment, where they are exposed to antibiotics, they usually start to mutate, and that is how they become multidrug-resistant pathogens [2, 3, 4].

Another thing to focus on is an explanation of the resistance principles as such and then in relation to enterococci. One of the most significant features of the *Enterococcus* genus’ adaptation is obtaining the genes responsible for resistance to vancomycin [3,4]. Enterococci are nowadays adapting even more and to other anti-gram-positive substances such as linezolid [5]. Recent problems stemming from the growing resistance of species *E. faecium* and nosocomial diseases caused by this bacterium are mentioned as well.

There is also not much information on the influence of enterococci on inflammatory bowel diseases such as ulcerative colitis and Crohn’s disease.

The aim of this review was to describe bacteria of the *Enterococcus* genus, their habitat, natural resistance, pathogenicity, and virulence. Another objective was to summarize the resistance formation and its importance in this genus focusing especially on the species *E. faecium*. The influences of enterococci on inflammatory bowel diseases and the following changes of intestinal microbiome were also described.

## 2 Characteristics of bacteria of the *Enterococcus* genus

### 2.1 Morphology

**Figure 1:**
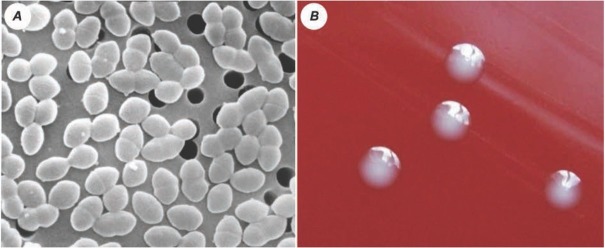
Enterococci cells, scanning electron microscope (*A*) [9] and the colonies of *Enterococcus faecalis*, blood agar, 24 hours cultivation, 37°C (*B*) [10]

Enterococci cells are gram-positive with an ovoid shape. They usually occur in pairs (Figure 1) or in chains of different lengths [1,4]. Enterococci form neither spores nor capsules, but some species may be capable of movement by the flagellum. These motile species are *Enterococcus casseliflavus* and *Enterococcus gallinarum*. Their colonies are milky white when grown on usual agar plates, but some species produce carotenoid pigments which color them yellow. Among these yellow colored enterococci are *E. sulfureus, E. casseliflavus* and *E. mundtii* [6, 7, 8].

### 2.2 Physiological and biochemical features

Enterococci belong to the group of gram-positive bacteria and most of the species of this genus have lysine-D-asparagin type of peptidoglycan except for *E. faecalis*, which has the lysine-alanin type [4, 11, 12, 13, 14, 15, 16]. The enterococci cell wall contains no proteins or lipids, but it has teichoic acid as a main surface antigen [17].

Temperature optimum ranges between 35 and 37°C, which is the average body temperature for mammals. Some species are capable of growth at a temperature of 45 °C or even at low temperatures around 10 °C. They can also adapt to 6,5% NaCl, and it is this adaptation, that allows them to further gain resistance to other factors such as a temperature of 62 °C, 22% ethanol or higher concentration of bile and sodium dodecyl sulphate. Enterococci are also very resistant to drying and therefore it is quite easy for them to persist in inappropriate conditions, which can be found for example in a hospital environment [4,18]. They are chemoorganotrophic, catalase negative and some species can show haemolytic activity. Complex media, rich with nutrients, are required for cultivation in laboratory conditions. Enterococci belong amongst facultative anaerobic bacteria with the preference of anaerobic environment; however, they do not mind living in aerobic conditions [8,19]. Survival in an environment with oxygen is provided by superoxide dismutase, which can transform toxic superoxide into less toxic peroxide and whose synthesis is induced by the presence of molecular oxygen in the air [20]. *Enterococci* are not capable of porphyrin synthesis; therefore, they lack cytochrome pigments and the ability to photosynthesize. However, some species may contain NADH peroxidases with flavin, which take part in fermentative metabolism, protect cells against oxidative stress within aerobic processes and can contribute to the bacterial virulence [21].

Enterococci have fermentative metabolism with homofermentative type. During this fermentation, glucose is being transformed to lactose, which is also the final product of this reaction. The process is called Embden-Meyerhof-Parnas pathway and it is actually a type of glycolysis. When in the environment with oxygen, glucose is metabolized to the acetic acid, acetoin, and carbon dioxide. Pyruvate is then metabolized according to the pH of the environment. In a mildly acid environment, at pH 5-6, pyruvate is transformed to lactate, while in the neutral or basic environment, pyruvate is metabolized to formate, ethanol and acetate in the ratio 2:1:1 [22]. Pyruvate is transformed into ethanol and acetate only when in the environment with a lack of nutrients [23].

### 2.3 Taxonomy

Even though the *Enterococcus* genus used to be classified as a D group streptococcus, it was separated from streptococci based on the results of DNA-DNA and DNA-rDNA hybridization studies [13]. Consequent 16S rRNA researches confirmed this division and on top of that, they showed differences among enterococci and the *Lactococcus* genus, which belongs to the family *Streptococcaceae* as well [24,25]. Nowadays, it is certain, that the *Enterococcus* genus belongs to the family *Enterococcaceae*, order *Lactobacillales*, class *Bacilli*, phylum *Firmicutes* and of course to the domain *Bacteria* [16]. There are 36 different species of enterococci known today, while 30 of them are divided into five groups according to their phylogenetic similarity: *E. faecalis*, *E. faecium*, *E. avium*, *E. gallinarum* a *E. cecorum*. Six species stand out of these groups [26]. This division is shown in the Table 1.

**Table 1:** Division of phylogenetically similar species of the *Enterococcus* genus [26]

E. faecium	E. avium	E. faecalis
*E. faecium*, *E. canis*,		
*E. durans*, *E. hirae*, *E. mundtii*, *E. ratti*, *E. villorum*, *E. asini*, *E. phoeniculicola*, *E. canintestini*, *E. thailandicus*	*E. avium*, *E. devriesei*, *E. gilvus*, *E. malodoratus*, *E. pseudoavium*, *E. raffinosus*, *E. pallens*, *E. hermanniensis*, *E. vikkiensis*	*E. faecalis*, *E. caccae*, *E. haemoperoxidus*, *E. moraviensis*, *E. silesiacus*, *E. termitis*

E. cecorum	E. gallinarum	Non-classified species
*E. cecorum*, *E. columbae*	*E. gallinarum*, *E. casseliflavus*	*E. aquimarinus*, *E. dispar*, *E. saccharolyticus*, *E. sulfureus*, *E. italicus*, *E. camelliae*

Outside of the official nomenclature, enterococci can be placed into the group of lactic acid bacteria (LAB). In this group they further belong to genera with content of G+C lower than 50% [27].

## 3 Occurrence of enterococci and their pathogenicity

### 3.1 Natural habitat

Enterococci survive in various environments due to their ability to easily adapt. They can be most often found in the soil, surface and waste waters, then in the gastrointestinal tract of birds, mammals, and even reptiles and insects. It can be found in fermented milk products as well, due to the fact that they belong to LAB [6]. Some species are capable of epiphytic life on plants. These species used to be mainly *E. mundtii* and *E. casseliflavus*, but more recent studies showed that *E. faecalis* and *E. faecium* belong to them as well [28].

Even greater diversity amongst species can be observed in several water sources. Quite new discoveries are *E. moraviensis* and *E. haemoperoxidus*, which persist in surface and waste waters or *E. aquimarinus*, which occurs in saltwater [6]. Their survival in saltwater is an amazing example of enterococci adaptation to their environment. It was discovered that some species can grow on zooplankton present in both fresh and saltwater, and therefore survive. Research has shown that the growing amount of plankton means a growing number of enterococci as well.

The amount of zooplankton, and therefore enterococci, is dependent on the water temperature, which leads to the highest occurrence during the summer months [29]. Enterococci were also found on a shore of freshwater Lake Michigan (USA) in dried algae of genus *Cladophora*, where they persisted for six months at the temperature of 4°C together with *Escherichia coli* [30].

The species *E. faecalis*, *E. faecium*, *E. durans*, and *E. hirae* can be found in surface and waste waters, coming probably from feces of animals and humans [6]. Intestinal bacteria, including enterococci can therefore be used for clean water testing where they function as so-called fecal indicators and their presence or higher concentration signifies fecal contamination in the water [29]. The most common enterococci occurring in the gastrointestinal tract are species *E. faecalis* and *E. faecium*, which are concurrently also the most common pathogens from the bacteria of *Enterococcus* genus [6].

### 3.2 Pathogenicity factors and virulence

Pathogenicity is described as the ability of a microorganism to cause an illness in several host species. This ability is determined by the pathogenicity factors such as transmissibility, toxicity and invasiveness. Quantitatively expressed pathogenicity for one strain is then called virulence. Pathogenicity factors are encoded in both chromosomal and plasmid genes. It is the presence of these genes in plasmids that enables the transmission of pathogenicity factors amongst strains and even species, leading to virulence increase in strains that were not that virulent once [31].

The species of enterococci living in the environment have quite low levels of virulence. However, the factors which can change the harmless strains into clinically problematic ones were found. Among these factors belongs mainly natural antibiotic resistance (clindamycin, cephalosporins, aminoglycosides), then the ability to gain and further spread plasmids coding other resistance (vancomycin) and finally a flexible genome able to adapt to different environments [1,6].

Enterococci begin their pathogenic process after cell adhesion to the host tissue, where they express genes for better adaptation to the environment with higher redox potential, lack of nutrients, phages and other defense mechanisms of the host. One of the factors that makes the host’s colonization easier is the presence of cytolysin or enterocin AS-48 in *E. faecalis* species. These substances are bactericidal to other cells and they kill their producent’s competition [32].

**Figure 2:**
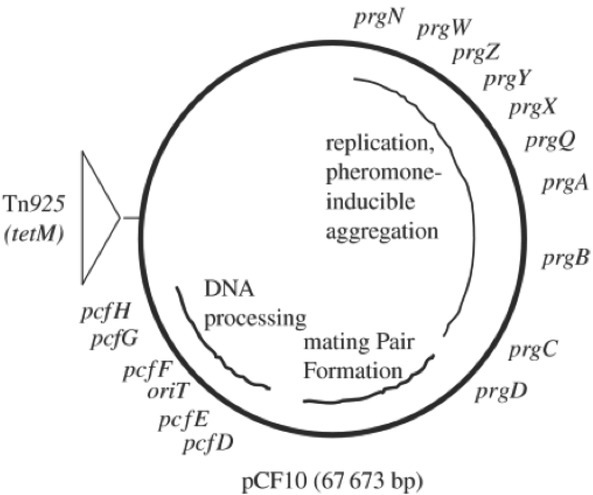
pCF10 plasmid map [34]

**Figure 3:**
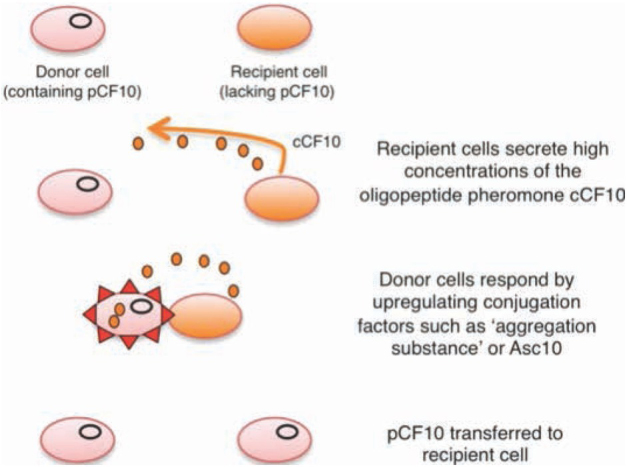
*E. faecalis* conjugation [35]

Another virulence factor is so-called aggregation substances (AS), which ease the plasmid transfer between two cells. Donor’s cell surface changes in the process, which enables the adhesion of potential recipient cells. Surface antigens of the donor cell play the biggest role in this process. That results in cluster formation, where the donor cell transmits a plasmid to the recipient one [32]. This process begins with the transmission of a chemical signal from the recipient cell to the donor one. This chemical signal called a pheromone is a short hydrophobic peptide, which induces an answer in the donor cells enabling aggregation. Most of the enterococci produce more types of these peptides, and they differ based on the type of plasmid in the donor cell. Certain peptides are therefore able to induce answer only in the cell carrying a certain plasmid [33]. As an example, the transfer of pCF10 plasmid carrying antibiotic resistance genes in bacterium *E. faecalis*, should be mentioned. These genes are located on the transposon TN*925* and one of them is *tetM* gene for the tetracycline resistance. The rest of the genes take care of the pheromone reaction and further aggregation, replication, DNA processing and formation of mating pair. To the pCF10 plasmid (Figure 2) then belongs heptapeptide cCF10 and mating them starts the expression of genes for conjugation (Figure 3) [34,35].

### 3.3 Epidemiology

Even though there are such a huge variety of enterococci in the environment, most of the epidemiological cases relate to nosocomial infections. The most common agents of these infections are species *E. faecalis* and *E. faecium*. *E. faecalis* used to be the number one agent in the past, but nowadays *E. faecium* is taking its role, especially for its growing antibiotic resistance [1,6]. Growth of enterococci in a hospital environment is also influenced by antibiotics used against gram-negative bacteria. This lowers the competition in the host organism and therefore provides advantages for gram-positive species, such as access to more nutrients and bigger growth space [1].

To the other factors making nosocomial transmission easier, belongs a long-term hospitalization, contact with infected patients and organs or bone marrow transplantation. Even though problems with VRE used to occur mostly in the hospitals throughout the USA in the past, their occurrence started to increase in European hospitals between 20^th^ and 21^th^ century, and to this day it is still rising [1].

Enterococci are common agents of nosocomial infections of soft tissues, which can lead to abscesses; urinary tract infections and endocarditis [32]. The urinary tract infections are usually connected with the use of catheters. In the case of abscesses, enterococci do not cause the infection by themselves, they usually work together with anaerobic microorganisms, which are otherwise avirulent. That means that these infections can be quite easily treated with antibiotics, which are effective only on anaerobic bacteria. By eliminating these microorganisms, they heal the infections caused mainly by enterococci as well [32].

## 4 Resistance formation and its genetic and molecular aspects

### 4.1 Mechanisms of resistance formation in bacteria of *Enterococcus* genus

Enterococci have an intrinsic resistance to some types of antibiotics and acquired resistance to the other ones. It is also necessary to mention that the resistant enterococci are mainly species *E. faecalis* and *E. faecium*, which belong to the clinically most significant ones. The most important antibiotics that enterococci express resistance to are β-lactam antibiotics (penicillin), cephalosporines, aminoglycosides, lincosamides and streptogramins. Moreover, they can also gain other mechanisms of resistance to β-lactams, aminoglycosides and streptogramins which leads to an increase of the original resistance, such as endurance to higher doses. Acquired resistance exists to glycopeptides (vancomycin), macrolides, tetracyclines, linezolid and chloramphenicol [5,36, 37, 38, 39, 40, 42].

### 4.2 β-lactam antibiotic and cephalosporines

β-lactam antibiotics and cephalosporines work on the same principle as pentapeptide precursors, which usually take part in the cell wall growth. These precursors bind onto enzymes, which are also called the penicillin binding proteins (PBPs), since they can form bonds with penicillin antibiotics as well. When this bond forms, cell wall synthesis is disrupted. This leads to the production of reactive oxygen species and cell lysis [5].

Enterococci have several mechanisms they use against this type of antibiotic. First is a genetically coded low affinity of the PBPs to β-lactams, which leads to the increase of minimum inhibitory concentration (MIC) in comparison to other related gram-positive bacteria [5]. Enterococci can also show tolerance to the β-lactams. Even though the cell wall synthesis is disrupted by high penicillin doses, superoxide dismutase produced by enterococci starts to remove the reactive oxygen species saving the cell from the lysis in the result [36].

The next resistance mechanism is an acquired one. It is provided by the *bla* genes which code β-lactamases production and can be transmitted via plasmids from one bacterium to another. β-lactamases disrupt β-lactam ring in the structure of the antibiotic and therefore strip them of their antimicrobial properties. Another type of acquired resistance is a formation of point mutations in *pbp5* genes, which occurs as a side effect after the usage of the antibiotics. These mutations lead to an extremely low affinity of PBP5 region to β-lactam antibiotics. Even though these mutations usually occur *de novo*, direct transfer of mutated *pbp5* genes was confirmed *in vitro* as well. This situation probably caused the wide spread of resistance in species *E. faecium* [5,37].

### 4.3 Aminoglycosides

Other types of antibiotics that this review was focused on are aminoglycosides, such as streptomycin and gentamicin. Aminoglycosides inhibit protein synthesis by binding irreversibly onto 30S subunit of the ribosome. To form this bond, antibiotic must first get into the cell by penetrating the cell wall. However, the enterococcal cell wall is naturally impermeable for aminoglycosides, which leads to the intrinsic resistance. It was discovered that this type of resistance can be easily overcome by using substances that disrupt cell wall synthesis, such as β-lactam antibiotics. Even though β-lactams do not induce the cell lysis by themselves, they weaken the cell wall enough for aminoglycosides to get inside the cell and work properly. The cooperation of two substances, when their effect together is bigger than the sum of their effects individually, is called synergy [38].

Other mechanisms of aminoglycoside resistance are acquired by plasmid transfer. These plasmids bear genes that code production of enzymes, which can inactivate the antibiotic and therefore make the binding onto 30S ribosomal subunit impossible. Strains with this type of acquired resistance are resilient even against the synergic therapy with the usage of antibiotics disrupting the cell wall synthesis. Moreover, enterococci are capable of transfer of these plasmids to other bacteria which leads to the spread of resistance. One of the genes located on these plasmids is gene *aac(6′)-Ie-aph(2″)-Ia*, which codes bifunctional enzyme Aac(6′)-Ie-Aph(2″)-Ia. The enzyme then takes care of phosphorylation of the second hydroxide group in the chemical structure of the antibiotic and inactivates its function. This gene is responsible for the huge increase of MIC in comparison to commonly used concentrations, as well as for acquired resistance to gentamicin and other structurally similar aminoglycosides except streptomycin. Streptomycin resistance can be caused by the presence of the enzymes Ant(6′)-Ia or Ant(3″)-Ia. These enzymes are adenylyltransferases and they inactivate the drug, which leads to the loss of its antimicrobial properties. The second type of streptomycin resistance is the occurrence of point mutations in the ribosome, which make the binding of streptomycin impossible and the protein synthesis therefore cannot be inhibited [38,39].

### 4.4 Lincosamides and streptogramins

The next resistance mechanisms are ion pumps, which remove the substance from the cell. They work especially against lincosamides and streptogramins - antibiotics, that inhibit protein synthesis by binding onto the subunit of 50S ribosome. Enterococci naturally express gene *lsa*, which is responsible for the function of ABC (ATP-binding cassette) pumps. This gene was observed only in species *E. faecalis* and it is responsible for resistance to clindamycin (lincosamide), quinupristin (class B streptogramin) and dalfopristin (class A streptogramin). A similar gene, *msrC*, exists in species *E. faecium*, but its ion pump provides resistance only to class B streptogramins.

There are three mechanisms of acquired resistance to lincosamides and streptogramins in enterococci. It can be either the acquisition of genes coding a new ion pump, acetylation leading to inactivation of the substance, or methylation of 23S rRNA leading to disruption of the binding site on the 50S ribosome subunit. The same mechanism may be the reason for the formation of macrolides resistance as well. Species *E. faecalis* was also discovered to have the ABC pump VgaD, it is coded by a plasmid and it provides resistance to class A streptogramins. The combination of this acquired resistance and the intrinsic one leads to the development of the strain resistant to class A and class B streptogramins, which results in resistance to combined medicaments such as quinupristin-dalfopristin, which is commonly used against vancomycin resistant enterococci. Methylation of the 50S subunit binding site is then provided by genes *ermA* and *ermB*, which are located on plasmids transmitted to the bacterium [5].

### 4.5 Glycopeptide antibiotics

**Figure 4:**
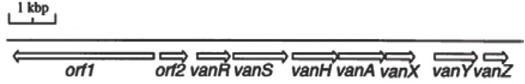
Genetic map of Tn*1546* transposon [41]

Solely acquired resistance in enterococci can be observed in relation to glycopeptide antibiotics such as vancomycin. These antibiotics were considered almost invincible and it was true for a long time. The first signs of resistance occurred 28 years after the introduction of vancomycin on the market. The effect of glycopeptides are completely different than the effect of other antibiotics. Vancomycin binds onto the D-ala-D-ala ending of the pentapeptide precursor in a peptidoglycan and inhibits the cell wall synthesis. Resistant strains can transform this pentapeptide by exchanging the ‘D-ala’ ending for ‘D-lac’ one. These modified precursors have a much lower affinity to glycopeptides in comparison with the original ones and they make it difficult for the antibiotic to bind onto peptidoglycan. This transformation is provided by transposon Tn*1546*, which codes several enzymes (Figure 4). Transposon Tn*1546* carries VanA operon, which is composed of two-component system vanR and VanS. This system further regulates gene expression for enzymes VanA, VanH, VanX, VanY and VanZ. When the system detects the presence of glycopeptide, it uses autophosphorylation to activate VanS. VanS further phosphorylates VanR, which interacts with specific promotors and increases the transcription of VanA, VanH, VanX, VanY and VanZ. At first VanH dehydrogenase transforms pyruvate to D-lactate and VanA ligase then binds D-ala and D-lac together. Enzymes of the host cell then bind D-ala-D-lac to the tripeptide precursor leaving the pentapeptide precursor unutilized. VanX further hydrolyses the D-ala-D-ala ending to amino acids in order to prevent it from being utilized in the cell wall synthesis. At last, VanY hydrolyses the remaining D-ala endings in non-transformed precursors in order to disrupt their function as well. This whole process creates new precursors with low affinity to glycopeptides and at the same time eliminates the unaltered precursors. VanZ enzyme also lowers susceptibility to teicoplanin, another glycopeptide antibiotic [5,40]

### 4.6 Linezolid

Another substance, which enterococci express resistance to, is linezolid. This antibiotic inhibits protein synthesis by binding to the initiation complex of 23S rRNA. Enterococci have more gene copies (4–6) coding this rRNA, therefore, resistance development used to be considered unlikely. Even though mutation occurs in one of the copies, the rest hides the mutated one and the 23S rRNA does not lose its binding site. The main issue occurs when susceptible cells recombine with the alternated ones. This recombination leads to the development of new strains with a bigger amount of mutated copies and, therefore, resistance to linezolid [5].

Gene *cfr* coding methyltransferase was also observed in enterococci. It alternates adenosine in the binding site of linezolid in 23S rRNA and prevents the binding of the antibiotic. This gene is located on plasmid pEF-01 and is considered transmittable. It was discovered for the first time in the *Staphylococcus* genus as a source of resistance to linezolid, lincosamides and class A streptogramins. However, linezolid resistance in enterococci is neither too common nor too significant [42].

Resistance to other antibiotics such as macrolides, tetracyclines or chloramphenicol is nowadays so common that the medication is chosen without these substances, therefore, the mechanisms of its formation do not have to be mentioned more thoroughly [5].

## 5 Spread prevention of antibiotic resistance

As was already mentioned, resistance developed mainly due to the usage of low doses of the antibiotic, which did not kill the microorganism, but let it adapt and then spread further. The principle of current resistance increase is still the same – the main issues are patients not finishing the full course of antibiotics, doctors prescribing antibiotics for non-bacterial diseases, and preventive administration of antibiotics to livestock. However, nowadays, the most significant contributor is a hospital environment. Bacteria living in hospitals are constantly in touch with low concentrations of antibiotics, which makes it easier for them to adapt. Resistance then can be transmitted between strains and in some cases even between species [43].

In this case, the most important criterion is proper hygiene from hospital staff as well as from patients and their visitors. Usage of disposable instruments such as catheters or gloves is nowadays a standard in developed countries; however, in order to improve prevention, it is also possible to implement for example single-use gowns for both nurses and visitors. Furthermore, non-disposable instruments such as stethoscopes should be assigned to infected patients only and in case of higher risk, isolation should be initiated. Patients with open wounds, patients with diarrhea and people with insufficient personal hygiene are considered a higher risk. The risk also rises in case of hospitalization of infected patients in the intensive care unit, in wards that use immunosuppressive treatment (hematology, oncology, transplantation units), or in neonatology wards. In case of hospitalization of a patient infected with resistant strains of enterococci, surfaces should be cleaned and disinfected more frequently, and suitable types of disinfection should be chosen. To sum up, the easiest possible solution at the moment is implementing precautions into hospital care to prevent further spread of resistance [44].

## 6 Vancomycin resistant *E. faecium*

### 6.1 Current problematic of growing resistance

Since its first occurrence in 1980, vancomycin resistance in the *Enterococcus* genus has spread massively. This can be observed most significantly in species *E. faecium*. The

**Figure 5:**
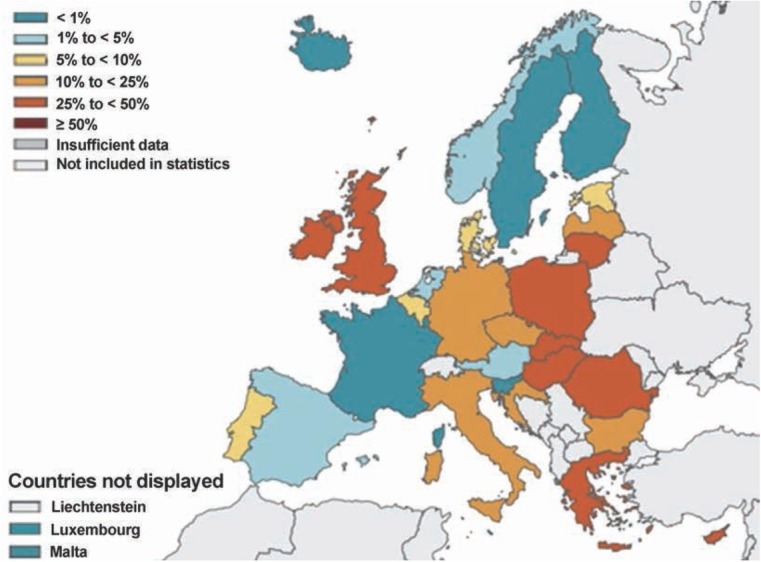
Map of Europe with a percentage of resistant strains from total number of isolates in species *E. faecium* in year 2017 [45]

EARS-Net statistics (The European Antimicrobial Resistance Surveillance Network) show an increase of vancomycin resistance in this species from 10, 4 % in year 2014 to 14, 9 % in year 2017 [45]. The current map of occurrence of the resistant strains in Europe is shown in Figure 5.

**Figure 6:**
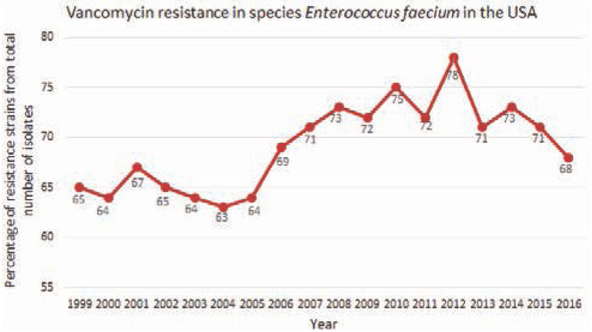
Graph of percentage of resistant strains of species *E. faecium* in the United States in years 1999–2016 [46]

A much worse situation can be observed in North America, especially in the United States. Even though the numbers are not growing much nowadays, the percentage is still one of the highest in the world. As shown in Figure 6, the percentage of resistant strains grew from 65 % in year 1999 to 68 % in the year 2016. The highest percentage can be seen in the year 2012, when it got up to 78 % [46].

**Figure 7:**
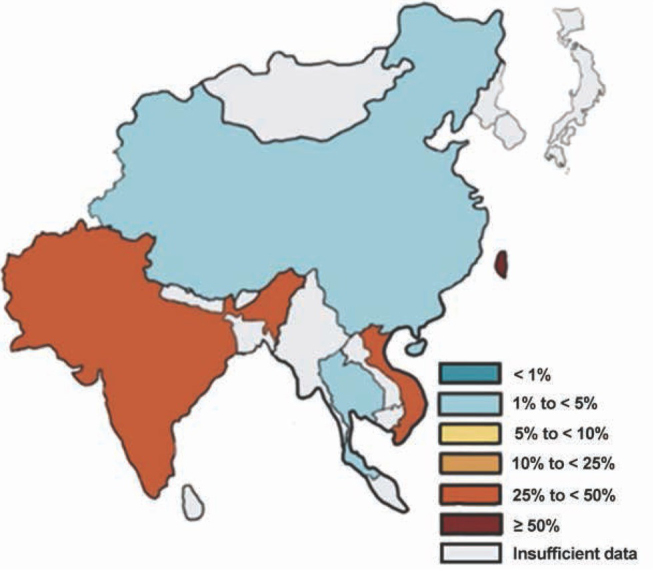
Map of Southeast Asia with a percentage of vancomycin resistant strains from the total number of isolates in species *E. faecium* in the year 2017 [46]

The situation in Asia is very similar to the one in Europe. Even though the percentage varies a lot amongst different states, almost nowhere were observed such high rates like in the USA. In total, 3 % of resistant isolates in China, 27 % in India, 4 % in Thailand, 27 % in Vietnam and high 66 % on the island Taiwan was detected. All these data have been collected between the years 2015-2017. In Australia, occurrence of resistance isolates reaches 50 %. Occurrence in Southeast Asia can be seen in a Figure 7 below, however, most of the countries do not keep statistics at all [46].

### 6.2 Nosocomial diseases linked to VREfm

As was already mentioned, *E. faecium* acquires resistance to most antibiotics much faster than other enterococci species and it is currently the most common enterococcus agent of nosocomial diseases. The most common nosocomial diseases caused by *E. faecium* are urinary tract infections and bloodstream infections, which can further develop into total sepsis of the organism. It can be often found in patients after surgeries, causing postoperative wounds infections, which can lead to the necessity of reoperation [47,48].

Another infection caused by *E. faecium* is endocarditis. Endocarditis is an inflammation of the heart valves and it occurs much more often in patients with implanted artificial valves [49]. The probability of occurrence also rises in immunocompromised patients, such as HIV positive people, people after transplantations or oncological patients. The correlation between infectious endocarditis and diabetes mellitus disease, liver cirrhosis, or dialysis also exists. While amongst vancomycin susceptible strains, the most common causative agent of endocarditis is *E. faecalis*, amongst vancomycin resistant strains it is *E. faecium*. However, this disease is not that common in comparison to other nosocomial infections caused by VREfm and it is not much explored [50]. Other diseases, non-nosocomial that could be possibly influenced by enterococci and other intestinal bacteria are inflammatory bowel diseases. To these diseases belong, for example, ulcerative colitis or Crohn’s disease and their exact origin is yet not known [51].

## 7 Enterococcal impact on inflammatory bowel diseases

### 7.1 Impact on ulcerative colitis

Ulcerative colitis (UC) is an autoimmune disease of the colon creating purulent inflammations and ulcers on the mucous and the submucous membrane of the rectum and large intestine [51, 52, 53, 54, 55, 56]. Patients often suffer from chronic and bloody diarrhoea and pains in lower abdomen. The correlation between ulcerative colitis and colorectal carcinoma was also observed. Even though, the cause of UC is not yet known, its origin is often linked to an unbalanced microbiome and reduced microbial diversity. Therefore, it is important to observe changes in the intestinal microbiome in patients with UC [57,58]. Moreover, an increased number of sulfate-reducing bacteria (SRB) is always detected in the patients with UC [59, 60, 61]. This intestinal bacterial group of microorganisms produces hydrogen sulfide in the process of dissimilatory sulfate reduction process. The production of hydrogen sulfide in the high concentration can inhibit other intestinal microbiome including lactic acid bacteria of *Lactobacillus* genus [62] and also their producers, SRB [63].

Past and present research shows that the number of enterococci (especially *E. faecalis*) in the gastrointestinal tract is significantly higher in patients with ulcerative colitis. The bigger diversity of the *Enterococcus* genus can be observed as well. Alterations of metabolism in bacteria isolated from ill people were also discovered. Strains isolated from patients demonstrated higher activity in mucin degradation and unlike the strains from healthy people, they were able to degrade hyaluronic acid. Both these substances serve as substrates for the lactic acid production. Due to the facts that intestines affected by ulcerative colitis produce more mucin than healthy intestines, and hyaluronic acid occurs in the ulcers, increased number of enterococci is likely caused by increased amount of substrate, which they can create lactic acid from and therefore thrive [64,65].

### 7.2 Impact on Crohn’s disease

Crohn’s disease (CD) is an inflammatory bowel disease as well. Unlike UC, inflammations caused by CD affect all layers of the colon from the oesophagus through the stomach to the rectum. While UC affects mainly the large intestine, inflammations caused by CD can be found rather in the small intestine. Inflamed and healthy sections of the intestine next to each other are typical for this disease. Same as the origin of UC, the origin of CD is not yet known, but it is being linked to intestinal microbiome and errors in immune response to common bacterial antigens [57].

Patients with Crohn’s disease have a higher number of enterococci in their intestines too, but it is not that high as in patients suffering from ulcerative colitis. Once more, we are talking mainly about the species *E. faecalis* [65]. Furthermore, virulent factors increase, especially the formation of aggregation substances, according to the changes of diseased intestinal wall. A sick intestine produces more fibronectin, which is related to the formation of AS. While the number of bacteria of AS negative strains and their adhesion is not significantly different in healthy people and in patients with CD, AS positive strains show much bigger adhesion to the intestinal mucosa leading to increased bacterial growth. The higher bacterial amount then leads to more extensive inflammation in the intestine and thereby to the worse progress of the disease [66,67].

To sum up, number of enterococci rises in both ulcerative colitis and Crohn’s disease. This is caused by changes on the intestinal wall, which provide an increased amount of substrate for the bacterial growth. Enterococcal population in patients with UC is also much more diverse and abundant than in patients with CD [65].

## 8 Conclusion

Described morphological, physiological and biochemical properties of bacteria of the *Enterococcus* genus as well as their taxonomy and its development throughout the history are important for better understanding of the resistance formation. Occurrence of the enterococci explained in detail allows us to understand the influence of the habitat on the pathogenicity and epidemiology of these bacteria. To better understand the resistance, it is essential to know the background of its historical development as well as the mechanisms of its formation to different types of antibiotics. The highest percentage of vancomycin resistant strains is then characteristic for the species *E. faecium*, and that is why this topic is so perspective. No less important is the possible impact of enterococci on inflammatory bowel diseases such as ulcerative colitis or Crohn’s disease. A connection between these diseases and enterococci may be crucial since their origin is still not yet known.

Enterococci occur mainly in the soil, surface and waste waters and in the gastrointestinal tract of animals and humans. They have a lot of virulence factors such as production of cytolysin for eradicating competitive bacteria or creation of aggregation substances. These factors help the bacteria to invade their host, develop a resistance to some types of antibiotics and spread it further.

Antibiotic resistance was discovered a long time ago and its occurrence still increases. Enterococci demonstrate resistance, both intrinsic and acquired, to a large number of medicaments such as β-lactam antibiotics, cephalosporines, aminoglycosides, lincosamides, streptogramins, glycopeptide antibiotics or linezolid. Resistance mechanisms often differ according to the type of the substance. The best prevention of its spreading is proper medication in enough doses, not using antibiotics in the livestock industry and avoiding the transfer of bacteria between hospitalized patients.

Currently, the most rapidly growing resistance can be found in vancomycin resistant *E. faecium*. Even though the highest occurrence of resistant strains can be found in the United States, it stays stable. A higher increase throughout the last years was recorded in Europe and some countries in Asia. These resistant strains are most often found in hospitals as causative agents of nosocomial diseases such as urinary tract or postoperative wound infections.

Enterococci are a part of the intestinal microbiome and thereby their impact on inflammatory bowel diseases should be researched. Patients with ulcerative colitis and Crohn’s disease have a larger number of enterococci in their intestines than healthy people. That is probably caused by changes in the intestinal wall according to ongoing inflammatory processes, which make it easier for bacteria to access nutrients and grow properly.

The resistance is still growing, and it is crucial to further research enterococci and try new antimicrobial therapies that might stop or slow down the resistance spread. It is also necessary to focus on their impact on inflammatory bowel diseases, since their origin is not yet fully recognized, and since the changes in the intestinal microbiome may be influenced by enterococci and we just do not have that knowledge yet.

## Abbreviations

AS: aggregation substance
CD: Crohn’s disease
CDDEP: Centre for Disease Dynamics, Economics & Policy
ECDC: European Centre for Disease Prevention and Control
LAB: lactic acid bacteria
MDR: multi drug resistant
MIC: minimum inhibitory concentration
MRSA: methicillin-resistant *Staphylococcus aureus*
PBPs: penicillin binding proteins
UC: ulcerative colitis
VRE: vancomycin-resistant enterococci
VREfm: vancomycin resistant *Enterococcus faecium*
